# Synthesis, Gastroprotective Effect and Cytotoxicity of New Amino Acid Diterpene Monoamides and Diamides †

**DOI:** 10.3390/molecules15107378

**Published:** 2010-10-21

**Authors:** Guillermo Schmeda-Hirschmann, Mariano Walter Pertino, Jaime A. Rodriguez, Francisco Monsalve, Daniel Droguett, Cristina Theoduloz

**Affiliations:** 1 Laboratorio de Química de Productos Naturales, Instituto de Química de Recursos Naturales, Universidad de Talca, Casilla 747, Talca, Chile; 2 Laboratorio de Cultivo Celular, Departamento de Bioquímica Clínica, Facultad de Ciencias de la Salud, Universidad de Talca, Talca, Chile; 3 Departamento de Ciencias Básicas, Facultad de Ciencias de la Salud, Universidad de Talca, Talca, Chile; 4 Departamento de Estomatología, Facultad de Ciencias de la Salud, Universidad de Talca, Talca, Chile

**Keywords:** labdane diterpenes, amino acid ester amides, gastroprotective effect, cytotoxicity

## Abstract

Following our studies on the gastroprotective effect and cytotoxicity of terpene derivatives, new amides were prepared from the diterpene 8(17)-labden-15,19-dioic acid (junicedric acid) and its 8(9)-en isomer with *C*-protected amino acids (amino acid esters). The new compounds were evaluated for their gastroprotective effect in the ethanol/HCl-induced gastric lesions model in mice, as well as for cytotoxicity using the following human cell lines: normal lung fibroblasts (MRC-5), gastric adenocarcinoma cells (AGS) and liver hepatocellular carcinoma (Hep G2). A dose-response experiment showed that at 25 mg/kg the C-15 leucyl and C-15,19-dileucylester amides of junicedric acid reduced gastric lesions by about 65.6 and 49.6%, respectively, with an effect comparable to lansoprazole at 20 mg/kg (79.3% lesion reduction). The comparison of the gastroprotective effect of 18 new amino acid ester amides was carried out at a single oral dose of 25 mg/kg. Several compounds presented a strong gastroprotective effect, reducing gastric lesions in the 70.9-87.8% range. The diprolyl derivative of junicedric acid, the most active product of this study (87.8% lesion reduction at 25 mg/kg) presented a cytotoxicity value comparable with that of the reference compound lansoprazole. The structure-activity relationships are discussed.

## 1. Introduction

Natural products, including plant drugs, have been used as gastroprotective agents all over the World. The natural products-derived therapeutic agents include the terpenes glycyrrhetic acid and sodium ecabet [1,2,3,4,5]. Several plant drugs are used in Latin American traditional medicine to treat symptoms related with gastric ulcers. The active gastroprotective constituents of some of the crude drugs are terpenes that present relevant activity in animal as well as in *in vitro* models [6,7,8,9,10,11,12,13,14,15,16,17]. 

It has been established that chronic gastric ulcers might lead to gastric and pancreatic cancer [18,19]. The resin of the tree *Araucaria araucana* is used to relieve gastric pain and the gastro-protective effect of the crude drug and main diterpene constituents has been reported [15]. The study started an investigation on the structure-activity relationships of labdane diterpenes from the resin for gastroprotective as well as for cytotoxic effect on normal and cancer cell lines [14,15,20]. Amide derivatives from labdane diterpenes with increased gastroprotective effect and low cytotoxicity were prepared [20]. However, the aromatic amines, used to prepare the amides, do not correspond to usual mammalian metabolites. Therefore, we selected amino acids esters to prepare new amides.

**Figure 1:**
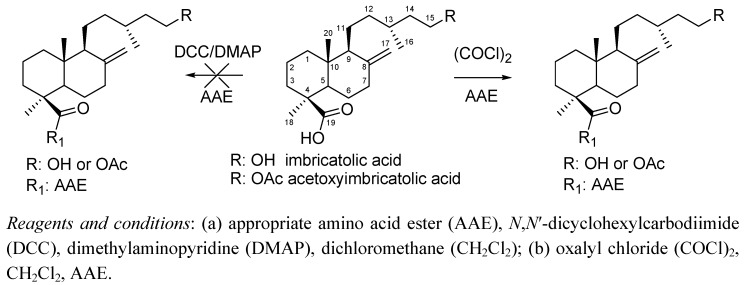
General procedure to prepare C-19 amides from imbricatolic acid and acetoxyimbricatolic acid as well as from the 8(9)-en isomers.

A previous study on the synthesis of C-19 amino acid ester monoamides was carried out using imbricatolic acid and its acetate, as well as the 8(9)-en isomers, as starting compounds (Figure 1). The new C-19 monoamides showed gastroprotective effects but the activity was lower than that found for amides of aromatic amines [21]. There is, however, no information on the gastroprotective effect of labdane diterpene amides bearing the amide at C-15, nor is the effect of C-15, 19-diamides on preventing gastric lesions or modulating cytotoxicity known. 

The aim of the present work was to synthesize new diterpene amides starting from the labdane diterpene imbricatolic acid. The starting compound was oxidized to obtain 8(17)-labden-15,19-dioic acid (junicedric acid) and then isomerized to the 8(9)-en derivative to prepare the new compounds. The new amides were assessed for gastroprotective effects in the ethanol/HCl-induced gastric lesions model in mice, as well as for cytotoxicity on normal human lung fibroblasts and the tumor cell lines AGS (gastric adenocarcinoma) and HepG2 (hepatocytes). The amino acids used to prepare the amides were *C*-methyl or ethyl esters of glycine, valine, leucine, proline and tryptophan. 

**Figure 2:**
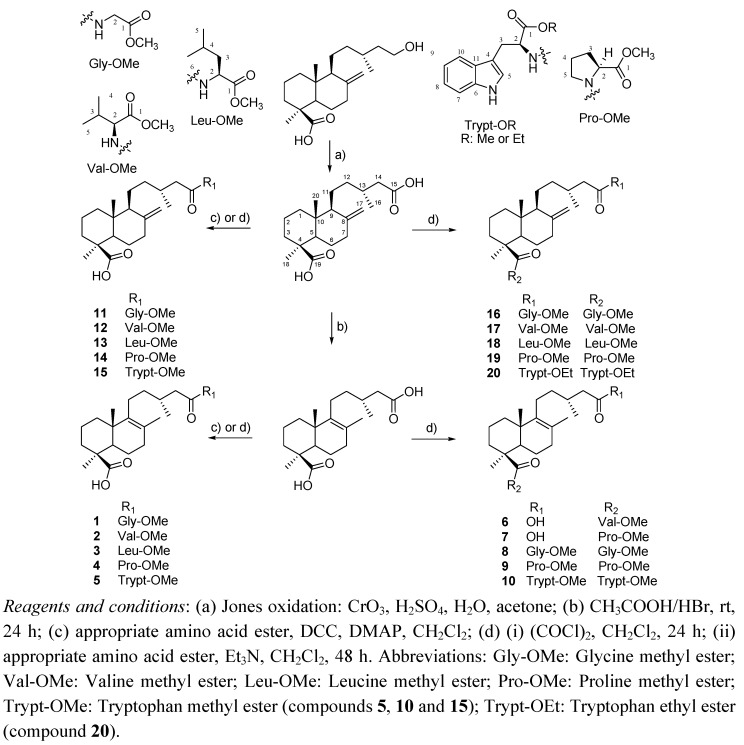
General procedure to prepare monoamides and diamides from dicarboxylic labdane diterpenes.

## 2. Results and Discussion

Starting from junicedric acid and its 8(9)-en isomer, 20 new diterpene amides were synthesized from amino acids esters and 18 from them were investigated for gastroprotective effects and cytotoxicity (Figure 2). 

To select the dose for comparing the gastroprotective effect of the new compounds, junicedric acid C-15 leucyl methyl ester amide (compound **13**) and C-15,19 dileucyl methyl ester amide (compound **18**) (amino acid COOH as methyl ester) were assessed as gastroprotective agents at three oral doses: 25, 50 and 100 mg/kg. In the dose-response study, the higher doses of both compounds reduced gastric lesions by 79-89% and did not differ statistically from each other. At 25 mg/kg, compound **13** reduced gastric lesions by 65.6% (lesion index in mm: 15.8 ± 6.2) and compound **18** by 49.6% (lesion index in mm: 23.2 ± 12.9). Under the same experimental conditions, the lesion index of untreated animals (in mm) was 46.0 ± 5.7 and the standard compound lansoprazole at 20 mg/kg reduced lesions by 79.3% (lesion index: 9.5 ± 2.5). Based on the results, the efficacy of the compounds preventing gastric lesions in mice was compared at the single oral dose of 25 mg/kg (Table 1). 

**Table 1:** Gastroprotective effect of the compounds **1**-**5**, **7**-**16**, **18**-**20** at 25 mg/kg on the HCl/EtOH induced gastric lesion model in mice.

Compound	Gastroprotective effect^a^	
	Lesion index	% Lesion reduction
**1**	12.3 ± 7.6**	73.3
**2**	19.1 ± 8.3*	58.5
**3**	12.6 ± 6.8**	72.6
**4**	13.4 ± 6.8**	70.9
**5**	34.3 ±12.0	25.4
**7**	21.2 ± 9.5*	53.9
**8**	17.5 ± 9.9**	62.0
**9**	18.4 ± 11.3**	60.0
**10**	17.6 ± 7.8**	61.7
**11**	12.9 ± 8.1**	71.9
**12**	15.4 ± 8.8**	66.5
**13**	15.8 ± 6.2**	65.6
**14**	18.5 ± 11.8*	59.8
**15**	12.1 ± 4.7**	73.7
**16**	12.2 ± 6.9**	73.5
**18**	23.2 ± 12.9*	49.6
**19**	5.6 ± 3.2**	87.8
**20**	14.3 ± 10.7*	68.9
Control	46.0 ± 5.7	0
Lansoprazole	9.5 ± 2.5**	79.3

^a^ Gastroprotective effect shown as mean lesion index ± standard deviation (SD) and percent lesion reduction compared with untreated controls. ^b^ Reference drug: lansoprazole at 20 mg/kg. Significance set at *P < 0.05; **P < 0.01.

For the junicedric acid 8(9)-en isomers, when the COOH function at C-19 was free, a higher gastroprotective activity was observed for the glycyl (**1**, 73.3%), leucyl (**3**, 72.6%) and prolyl ester (**4**, 70.9%) amides, which were more effective as gastroprotective agents than the corresponding C-15,19-diamides. However, for the tryptophanyl ester derivatives, better gastroprotection was found for the diamide **10** (61.7%) than for the monoamide **5** (25.4%), with lower cytotoxicity seen for compound **10**. For the diamides with glycine, proline and tryptophan ester, better gastroprotection was observed for the derivatives with the *exo* methylene group.

Under our experimental conditions and at a single oral dose of 25 mg/kg, the best gastroprotective effect measured as percent lesion reduction was observed for compounds **1 **(73.3%), **3 **(72.6%), **4 **(70.9%), **11 **(71.9%), **15 **(73.7%), **16** (73.5%) and **19 **(87.8%). Compounds **12 **(66.5%), **13** (65.6%), and **20 **(68.9%) also presented good activity. Among the amide derivatives of junicedric acid with a free COOH function at C-19 (compounds **11**-**15**), higher lesion reduction was observed for compounds **11** (71.9%), **15** (73.7%) and **16** (73.5%) while for the C-15,19 diamides the proline ester derivative **19** (87.8%) and the tryptophan ester derivative **20** (68.9%) showed better gastroprotective effects. 

When considering cytotoxicity (Table 2), significant differences can be observed between the compounds. Lower cytotoxicity with IC_50_ values > 1,000 µM towards all cell lines was observed for the tryptophan ester monoamide **15** and the diamides **10** and **20**, making these derivatives the most interesting compounds when looking for gastroprotective products with low cell toxicity. 

**Table 2:** Cytotoxicity (IC_50 _values, µM) of the compounds **1**-**5**, **7**-**16**, **18**-**20**.

Compound	Cytotoxicity^a^ IC_50 _(µM)^b^		
	MRC-5	AGS	Hep G2
**1**	447 ± 26	189 ± 9	242 ± 13
**2**	> 1,000	> 1,000	> 1,000
**3**	> 1,000	445 ± 27	806 ± 32
**4**	288 ± 11	167 ± 7	321 ± 14
**5**	260 ± 15	149 ± 7	291 ± 11
**7**	459 ± 32	266 ± 16	364 ± 15
**8**	890 ± 45	592 ± 30	841 ± 42
**9**	119 ± 7	82 ± 4	67 ± 3
**10**	> 1,000	> 1,000	> 1,000
**11**	> 1,000	361 ± 18	541 ± 22
**12**	380 ± 19	309 ± 18	673 ± 34
**13**	449 ± 29	308 ± 17	808 ± 48
**14**	> 1,000	297 ± 14	894 ± 56
**15**	> 1,000	> 1,000	> 1,000
**16**	> 1,000	483 ± 29	529 ± 36
**18**	495 ± 35	> 1000	> 1000
**19**	267 ± 14	206 ± 10	316 ± 16
**20**	> 1,000	> 1,000	> 1,000
Lansoprazole	306 ± 11	162 ± 6	221 ± 9

^a ^Cultured human cell lines: MRC-5, human normal lung fibroblasts; AGS, gastric epithelial adenocarcinoma cells; Hep G2, hepatocytes. ^b ^Values are arithmetic means of three different experiments in quadruplicate ± SD. Confluent cultures were treated with the culture medium containing the compounds at concentrations ranging between 0 and 1000 µM for 24 h. Cell viability was determined by the neutral red uptake assay. ^c^ Lansoprazole was used as the reference drug.

Compound **9** proved to be the more cytotoxic derivative, with IC_50_ values of 119, 82 and 67 µM against human fibroblasts, AGS cells and Hep G2 hepatocytes, respectively. None of the new compounds presented selectivity against the cell panel used, but higher gastroprotective effect with lower toxicity was found for compounds **15** and **20**. Compound **19**, the most active product of this study, presented a cytotoxicity comparable with that of the reference compound lansoprazole, but higher than that of compounds **15** and **20**, which presented IC_50_ values > 1,000 µM. 

## 3. Experimental

### 3.1. General

Optical rotations were obtained for solutions in CHCl_3_ (concentrations expressed in g/100 mL) on a Jasco DIP 370 polarimeter. Infrared spectra were recorded on a Nicolet Nexus FT-IR instrument (Thermo Electron Corporation). NMR spectra were recorded at room temperature in CDCl_3_ using a Bruker Avance 400 instrument (Bruker, Germany) operating at 400 MHz for ^1^H and 100 MHz for ^13^C spectra. All chemical shifts values are reported relative to residual CHCl_3_.Chemical shifts (δ) are given in ppm and coupling constants (J) are reported in Hertz. Mass spectra were measured with an EBE trisector VG Autospec Micromass spectrometer operating at 70 eV and are presented as m/z (rel. int. %). Silica gel 60 (Merck, 63-200 µm particle size) was used for column chromatography, precoated silica gel plates (Merck, Kieselgel 60 F_254_, 0.25 mm) were used for thin layer chromatography (TLC) analysis. Reversed-phase silica gel 100 C_8_ (Fluka, Germany) was used to purify the the amides. TLC spots were visualized by spraying the chromatograms with p-anisaldehyde-ethanol-acetic acid-H_2_SO_4_ (2:170:20:10 v/v) and heating at 110 ºC for 3 min. 

### 3.2. Synthesis of the diterpene amides *1-20*

The diterpene used as starting compound for the synthesis was obtained from the resin of *Araucaria araucana* as described in previous work [14,15]. 15-Hydroxyimbricatolic acid was treated with Jones reagent (CrO_3_/H_2_SO_4_/H_2_O) to afford after purification by column chromatography on silica gel the diacid diterpene junicedric acid. The 8(9)-en junicedric acid isomer was prepared treating junicedric acid with HBr in acetic acid. The amides described herein for the first time were prepared under an inert (N_2_) atmosphere. Briefly, the diterpene was dissolved in dry dichloromethane (CH_2_Cl_2_, DCM) and ice cooled under nitrogen flow. To this solution, oxalyl chloride in dry DCM was added dropwise with stirring. The volume of dry DCM used to dissolve the diterpene was about 15-20 mL. Oxalyl chloride was dissolved in dry DCM in a 1:5 ratio The diterpene:oxalyl chloride molar ratio was 1:40. The mixture was stirred at room temperature overnight, then the DCM was evaporated and the residue vacuum dried. The dry residue was dissolved in dry DCM and the *C*-protected amino acid ester (AAE) (as hydrochloride) was added as well as triethylamine (TEA) under constant N_2_ flow. The diterpene:AAE:TEA molar ratio was 1:3:3. The reaction mixture was left at room temperature (18-20 ºC) under stirring and inert atmosphere for two days (48 h). Then, the mixture was washed two times with water, and the aqueous phase extracted with DCM to obtain the crude reaction mixture [21,22]. To prepare the C-15 monoamides, the diterpene dissolved in dry DCM was treated with *N*,*N*′-dicyclohexylcarbodiimide (DCC)/dimethylaminopyridine (DMAP) and the amino acid ester.HCl. The reaction time was 1-3 h and the process was monitored by TLC. Purification was undertaken using a combination of gel permeation in Sephadex LH-20, eluting with an petroleum ether (PE):DCM:MeOH 1:1:1 mixture, silica gel column chromatography (CC) and reversed-phase silica gel chromatography (RP-8) to afford the amides. The reagents and conditions used are summarized in Figure 2. The structure of the compounds was determined mainly by NMR spectroscopy and by comparing the spectroscopic data with those of similar compounds already reported and by IR and mass spectra. 

*Labd-8(9)-en-15,19-dioic acid, 15-glycyl methyl ester amide* (**1**). The diterpene diacid (0.288 g, 0.857 mmol) was treated with oxalyl chloride to yield the acid chloride who was treated with glycine methyl ester.HCl)/TEA to afford after 24 h compound **1 **(0.266 g, 0.653 mmol, 76.2% yield, Rf 0.08, PE:EtOAc 6:4) as a colourless resin. Using DCC/DMAP, we did not obtain the desired product. [*α*]_D_^20^: +45.5 (*c* 0.176, CHCl_3_); IR *ν*_max_ (film) 3,311, 2,952, 2,872, 1,792, 1,748, 1,653, 1,537, 1,433, 1,202, 991, 748 cm^-1^; ^1^H-NMR (CDCl_3_) δ 0.94 (3H, s, H-20), 1.01 (3H, d, *J =* 6.4 Hz, H-16), 1.32 (3H, s, H-18), 1.59 (3H, s, H-17), 3.80 (3H, s), 4.05 (1H, dd, *J =* 18.8, 5.2 Hz, Gly H-2), 4.10 (1H, dd, *J = *18.8, 5.2 Hz, Gly H-2), 5.98 (1H, dd *J = *4.8, 4.8 Hz, NH); ^13^C-NMR (CDCl_3_): 37.63 (t, C-1), 19.64 (t, C-2), 38.09 (t, C-3), 45.64 (s, C-4), 53.99 (d, C-5), 21.14 (t, C-6), 37.34 (t, C-7), 126.63 (s, C-8), 139.56 (s, C-9), 39.87 (s, C-10), 25.90 (t, C-11), 34.52 (t, C-12), 31.99 (d, C-13), 44.17 (t, C-14), 173.55 (s, C-15), 19.87 (q, C-16), 19.64 (q, C-17), 27.99 (q, C-18), 170.78 (s, C-19), 19.06 (q, C-20); Gly: 172.84 (s, C-1’), 41.40 (t, C-2’); 52.57 (q, OMe); ESI-MS *(m/z)*: 430.0926. Calcd for [C_23_H_37_NO_5_Na^+^]: 430.2570. 

*Labd-8(9)-en-15,19-dioic acid, 15-valyl methyl ester amide* (**2**). Some 0.457 g of the diterpene diacid (1.36 mmol) afforded after treatment with DCC/DMAP and valine methyl ester.HCl, the compound **2** (0.242 g, 39% yield, Rf 0.33, PE:EtOAc 6:4) as a colourless resin. [*α*]_D_^20^: +52.8 (*c* 0.843, CHCl_3_); IR *ν*_max_ (film) 3,334, 2,956, 2,932, 2,872, 1,796, 1,740, 1,649, 1,537, 1,465, 1,437, 1,382, 1,206, 995, 756 cm^-1^; ^1^H-NMR (CDCl_3_) δ 0.88 (3H, s, H-20), 0.91 (6H, d, *J = *6.8 Hz, Val H-4, H-5), 0.95 (3H, d, *J =* 6.4 Hz, H-16), 1.27 (3H, s, H-18), 1.53 (3H, s, H-17), 2.16-2.18 (1H, m, Val H-3), 3.71 (3H, s), 4.56 (1H, dd, *J =* 8.8, 4.8 Hz, Val H-2), 5.87 (1H, d, *J = *8.8 Hz, NH); ^13^C-NMR (CDCl_3_): 37.50 (t, C-1), 19.40 (t, C-2), 37.90 (t, C-3), 45.44 (s, C-4), 53.82 (d, C-5), 20.95 (t, C-6), 37.16 (t,C-7), 126.39 (s, C-8), 139.37 (s, C-9), 39.69 (s, C-10), 25.69 (t, C-11), 34.33 (t, C-12), 31.86 (d, C-13), 44.37 (t, C-14), 172.74 (s, C-15), 19.48 (q, C-16), 19.70 (q, C-17), 27.81 (q, C-18), 173.34 (s, C-19), 18.96 (q, C-20); Val: 172.37 (s, C-1’), 56.88 (d, C-2’), 31.33 (d, C-3’), 18.88 (q, C-4’), 17.90 (q, C-5’); 52.12 (q, OMe); ESI-MS *(m/z)*: 472.6656. Calcd for [C_26_H_43_NO_5_Na^+^]: 472.3039. 

*Labd-8(9)-en-15,19-dioic acid, 15-leucyl methyl ester amide* (**3**). Treatment of the diacid (0.401 g, 1.19 mmol) with DCC/DMAP and leucine methyl ester.HCl afforded **3** (0.313 g, 0.67 mmol, 56.7 % yield, Rf 0.28, PE:EtOAc 6:4) as a colourless resin. [*α*]_D_^20^: +55.33 (*c* 0.459, CHCl_3_); IR *ν*_max_ (film) 3,307, 2,952, 2,932, 2,868, 1,796, 1,744, 1,649, 1,537, 1,378, 995, 756 cm^-1^; ^1^H-NMR (CDCl_3_) δ 0.94 (3H, s, H-20), 0.96 (3H, d, *J =* 6.0 Hz, Leu H-6), 0.97 (3H, d, *J = *6.0 Hz, Leu H-5), 1.00 (3H, d, *J =* 5.6 Hz, H-16), 1.32 (3H, s, H-18), 1.59 (3H, s, H-17), 3.76 (3H, s), 4.69 (1H, ddd, *J = *5.2, 8.6, 8.8 Hz, Leu H-2), 5.83 (1H, br d, *J = *5.6 Hz, NH); ^13^C-NMR (CDCl_3_): 37.66 (t, C-1), 19.60 (t, C-2), 38.07 (t, C-3), 45.60 (s, C-4), 53.99 (d, C-5), 21.11 (t, C-6), 37.34 (t, C-7), 126.52 (s, C-8), 139.58 (s, C-9), 39.84 (s, C-10), 26.19 (t, C-11), 34.50 (t, C-12), 32.00 (d, C-13), 44.40 (t, C-14), 172.42 (s, C-15), 23.00 (q, C-16), 19.83 (q, C-17), 27.97 (q, C-18), 173.88 (s, C-19), 19.04 (q, C-20); Leu: 173.47 (s, C-1’), 50.72 (d, C-2’), 41.98 (t, C-3’), 22.11 (d, C-4’), 19.61 (q, C-5’), 19.61 (q, C-6’); 52.38 (q, OMe); ESI-MS *(m/z)*: 486.3339. Calcd for [C_27_H_45_NO_5_Na^+^]: 486.3195. 

*Labd-8(9)-en-15,19-dioic acid, 15-prolyl methyl ester amide* (**4**). Treatment of the diacid (331 mg, 0.985 mmol) with DCC/DMAP and proline methyl ester.HCl yielded compound **4** (55.2 mg, 0.123 mmol, 12.5 % yield, Rf 0.10, PE:EtOAc 6:4) as colourless resins. [*α*]_D_^20^: +34.24 (*c *0.552, CHCl_3_); IR *ν*_max_ (film) 3,454, 2,948, 2,872, 1,796, 1,748, 1,641, 1,429, 1,413, 1,374, 1,198, 1,178, 991, 748 cm^-1^;^ 1^H-NMR (CDCl_3_) δ 0.93 (3H, s, H-20), 1.01 (3H, d, *J =* 6.4 Hz, H-16), 1.31 (3H, s, H-18), 1.59 (3H, s, H-17), 3.53 (1H, m, Pro H-5), 3.67 (1H, m, Pro H-5), 3.74 (3H, s), 4.50 (1H, dd, *J = *8.0, 3.2 Hz, Pro H-2); ^13^C-NMR (CDCl_3_): 37.76 (t, C-1), 19.55 (t, C-2), 38.06 (t, C-3), 45.59 (s, C-4), 53.98 (d, C-5), 21.11 (t, C-6), 34.49 (t, C-7), 126.46 (s, C-8), 139.65 (s, C-9), 39.83 (s, C-10), 25.80 (t, C-11), 37.31 (t, C-12), 31.43 (d, C-13), 41.73 (t, C-14), 171.71 (s, C-15), 19.71 (q, C-16), 19.85 (q, C-17), 27.94 (q, C-18), 173.49 (s, C-19), 19.04 (q, C-20); Pro: 173.10 (s, C-1’); 58.78 (d, C-2’); 29.37 (t, C-3’); 24.98 (t, C-4’); 47.39 (t, C-5’); 52.24 (q, OMe); ESI-MS *(m/z)*: 470.1352. Calcd for [C_26_H_41_NO_5_Na^+^]: 470.2882. 

*Labd-8(9)-en-15,19-dioic acid, 15-tryptophanyl methyl ester amide* (**5**). Some 483 mg of the diacid (1.44 mmol) was treated with DCC/DMAP and tryptophane methyl ester.HCl to afford **5** as a colourless resin (62 mg, 0.116 mmol, 8% yield, Rf 0.21, PE:EtOAc 6:4). [*α*]_D_^20^: +0.81 (*c* 0.618, CHCl_3_); IR *ν*_max_ (film) 3,314, 2,928, 2,852, 1,736, 1,692, 1,645, 1,557, 1,517, 1,453, 1,437, 1,378, 1,218, 756 cm^-1^; ^1^H-NMR (CDCl_3_) δ 0.82 (3H, s, H-20), 0.86 (3H, d, *J = *6.0 Hz, H-16), 1.24 (3H, s, H-18), 1.51 (3H, s, H-17), 3.28 (2H, m, Tript H-3), 3.66 (3H, s), 4.96 (1H, m, Tript H-2), 5.99 (1H, br d, *J = *7.2 Hz, Tript NH), 6.95 (1H, br s, Tript H-5), 7.08 (1H, dd, *J = *7.8, 7.1 Hz, Tript H-8), 7.16 (1H, dd, *J =* 7.5, 7.2 Hz, Tript H-9), 7.32 (1H, d, *J = *8.1 Hz, Tript H-7), 7.50 (1H, d, *J = *8.2 Hz, Tript H-10), 8.28 (1H, br s, NH); ^13^C-NMR (CDCl_3_): 37.67 (t, C-1), 19.72 (t, C-2), 37.75 (t, C-3), 43.94 (s, C-4), 53.79 (d, C-5), 20.95 (t, C-6), 37.37 (t, C-7), 126.77 (s, C-8), 139.39 (s, C-9), 39.96 (s, C-10), 25.03 (t, C-11), 34.45 (t, C-12), 31.94 (d, C-13), 44.24 (t, C-14), 172.64 (s, C-15), 19.56 (q, C-16), 19.91 (q, C-17), 28.82 (q, C-18), 183.68 (s, C-19), 18.14 (q, C-20); Trypt: 172.80 (s, C-1’); 53.06 (d, C-2’); 27.91 (t, C-3’); 110.25 (s, C-4’); 122.91 (d, C-5’); 136.34 (s, C-6’); 111.51 (d, C-7’); 119.86 (d, C-8’); 122.43 (d, C-9’); 118.73 (d, C-10’); 127.90 (s, C-11’); 52.53 (q, OMe); ESI-MS *(m/z)*: 559.3320. Calcd for [C_32_H_44_N_2_O_5_Na^+^]: 559.3250. 

*Labd-8(9)-en-15,19-dioic acid, 19-valyl methyl ester amide* (**6**). The diacid (210 mg. 0.61 mmol) was treated with oxalyl chloride to afford the acid chloride. After reaction with valine methyl ester.HCl/TEA and purification, the compound **6** was obtained as a colourless resin (57 mg, 0.127 mmol, 21% yield, Rf: 0.23 (PE:EtOAc 7:3). [*α*]_D_^20^: +70 (*c* 0.057, CHCl_3_); IR *ν*_max_ (film) 3318, 2960, 2932, 2880, 1740, 1724, 1692, 1641, 1537, 1210, 1154, 748 cm^-1^; ^1^H NMR (CDCl_3_) δ 0.80 (3H, s, H-20), 0.87 (6H, d, *J =* 6.9 Hz, Val H-4, H-5), 0.91 (3H, d, *J = *6.4 Hz, H-16), 1.19 (3H, s, H-18), 1.50 (3H, s, H-17), 3.69 (3H, s), 4.56 (1H, dd, *J =* 8.8, 5.0 Hz, Val H-2), 6.14 (1H, d, *J = *8.8 Hz, NH); ^13^C NMR (CDCl_3_): 37.63 (t, C-1), 19.69 (t, C-2), 37.67 (t, C-3), 43.91 (s, C-4), 53.71 (d, C-5), 20.91 (t, C-6), 37.36 (t, C-7), 126.71 (s, C-8), 139.36 (s, C-9), 39.89 (s, C-10), 25.83 (t, C-11), 34.41 (t, C-12), 32.01 (d, C-13), 44.37 (t, C-14), 173.12 (s, C-15), 19.59 (q, C-16), 19.88 (q, C-17), 28.82 (q, C-18), 183.64 (s, C-19), 19.09 (q, C-20); Val: 172.89 (s, C-1’), 57.04 (d, C-2’), 31.45 (d, C-3’), 18.14 (q, C-4’), 18.05 (q, C-5’); 52.31 (q, OMe); ESI-MS *(m/z)*: 472.3318. Calcd for [C_26_H_43_NO_5_Na^+^]: 472.3039. 

*Labd-8(9)-en-15,19-dioic acid, 19-prolyl methyl ester amide* (**7**). For preparation please see under compound **9**. Colorless resin; [*α*]_D_^20^: +26.0 (*c *1.02, CHCl_3_); IR *ν*_max_ (film) 3,420, 2,952, 2,876, 1,792, 1,740, 1,641, 1,437, 1,198, 1,174, 991, 756 cm^-1^; ^1^H-NMR (CDCl_3_) δ 0.81 (3H, s, H-20), 0.95 (3H, d, *J = *6.4 Hz, H-16), 1.20 (3H, s, H-18), 1.52 (3H, s, H-17), 3.49 (1H, m, Pro H-5), 3.62 (1H, m, Pro H-5), 3.67 (3H, s), 4.46 (1H, dd, *J = *8.4, 3.6 Hz, Pro H-2); ^13^C-NMR (CDCl_3_): 37.48 (t, C-1), 19.51 (t, C-2), 37.68 (t, C-3), 43.77 (s, C-4), 53.49 (d, C-5), 20.76 (t, C-6), 34.27 (t, C-7), 126.51 (s, C-8), 139.34 (s, C-9), 39.75 (s, C-10), 25.70 (t, C-11), 37.20 (t, C-12), 31.36 (d, C-13), 41.57 (t, C-14), 171.84 (s, C-15), 19.51 (q, C-16), 19.74 (q, C-17), 28.68 (q, C-18), 183.64 (s, C-19), 17.98 (q, C-20); Pro: 172.96 (s, C-1’); 58.64 (d, C-2’); 29.23 (t, C-3’); 24.81 (t, C-4’); 47.29 (t, C-5’); 52.14 (q, OMe); ESI-MS *(m/z)*: 470.1352. Calcd for [C_26_H_41_NO_5_Na^+^]: 470.2882. 

*Labd-8(9)-en-15,19-dioic acid, 15,19-diglycyl methyl ester amide* (**8**). The diacid (203 mg, 0.604 mmol) was treated with oxalyl chloride to afford the acid chloride. After reaction with glycine methyl ester.HCl/TEA and purification on Sephadex LH-20, the compound **8** was obtained as a colourless resin. (68 mg, 0.142 mmol, 24% yield, Rf: 0.20 (PE:EtOAc 1:1). [*α*]_D_^20^: +69 (*c* 0.056, CHCl_3_); IR *ν*_max _(film) 3,382, 3,334, 2,944, 2,860, 1,752, 1,645, 1,521, 1,437, 1,366, 1,206, 752 cm^-1^; ^1^H-NMR (CDCl_3_) δ 0.80 (3H, s, H-20), 0.94 (3H, d, *J =* 6.3 Hz, H-16), 1.18 (3H, s, H-18), 1.53 (3H, s, H-17), 3.74 (6H, s), 3.97 (2H, m, Gly H-2), 4.05 (2H, m, Gly H-2), 6.11 (1H, t, *J =* 4.8 Hz, NH), 6.16 (1H, t, *J = *4.8 Hz, NH); ^13^C-NMR (CDCl_3_): 37.70 (t, C-1), 19.83 (t, C-2), 38.06 (t, C-3), 44.03 (s, C-4), 53.90 (d, C-5), 21.41 (t, C-6), 37.59 (t, C-7), 126.40 (s, C-8), 139.61 (s, C-9), 39.92 (s, C-10), 25.84 (t, C-11), 34.65 (t, C-12), 31.94 (d, C-13), 44.03 (t, C-14), 172.88 (s, C-15), 19.56 (q, C-16), 19.85 (q, C-17), 29.76 (q, C-18), 177.38 (s, C-19), 18.17 (q, C-20); Gly: 171.03, 170.71 (s, C’-1), 41.45, 41.31 (t, C’-2); 52.36, 52.31 (q, OMe); ESI-MS *(m/z)*: 501.3414. Calcd for [C_26_H_42_N_2_O_6_Na^+^]: 501.2941. 

*Labd-8(9)-en-15,19-dioic acid, 15,19-diprolyl methyl ester amide *(**9**). Treatment of the diacid (0.289 g, 0.86 mmol) with oxalyl chloride afforded the acid chloride which was treated with proline methyl ester.HCl /TEA to afford the monoamide **7** (90 mg, 0.2 mmol, 23% yield, Rf 0.33, PE:EtOAc 1:1) and the diamide **9 **(55 mg, 0.098 mmol, 11.4% yield, Rf 0.53, PE:EtOAc 1:1) as colourless resins. Compound **9**: [*α*]_D_^20^: +29.0 (CHCl_3_; c = 0.558); IR *ν*_max_ (film) 3370, 2948, 2880, 1744, 1720, 1649, 1433, 1194, 752 cm^-1^; ^1^H-NMR (CDCl_3_) δ 0.76 (3H, s, H-20), 1.00 (3H, d, *J =* 6.4 Hz, H-16), 1.20 (3H, s, H-18), 1.58 (3H, s, H-17), 3.53 (1H, m, Pro H-5), 3.66 (1H, m, Pro H-5), 3.64 (3H, s), 3.73 (3H, s), 4.51 (1H, dd, *J =* 8.8, 2.8 Hz, Pro H-2); ^13^C-NMR (CDCl_3_): 37.84 (t, C-1), 19.78 (t, C-2), 37.94 (t, C-3), 44.07 (s, C-4), 53.79 (d, C-5), 21.05 (t, C-6), 34.50 (t, C-7), 126.66 (s, C-8), 139.56 (s, C-9), 39.73 (s, C-10), 25.85 (t, C-11), 37.42 (t, C-12), 31.48 (d, C-13), 41.77 (t, C-14), 171.78 (s, C-15), 19.71 (q, C-16), 19.91 (q, C-17), 28.62 (q, C-18), 178.28 (s, C-19), 17.95 (q, C-20); Pro: 173.12 (s, 2 C, C-1’); 58.78 (d, 2 C, C-2’); 29.38 (t, 2 C, C-3’); 24.99 (t, 2 C, C-4’); 47.40 (t, 2 C, C-5’); 52.26 (q, OMe) 51.22 (q, OMe); ESI-MS *(m/z)*: 581.4495. Calcd for [C_32_H_50_N_2_O_6_Na^+^]: 581.3566.

*Labd-8(9)-en-15,19-dioic acid, 15,19-ditryptophanyl methyl ester amide* (**10**). Some 204 mg of the diacid (0.607 mmol) was treated with oxalyl chloride to obtain the acid chloride and reacted with tryptophane methyl ester.HCl/TEA. After purification by CC on silica gel, compound **10** was obtained as a colourless resin (259 mg, 0.352 mmol, 58% yield). [*α*]_D_^20^: +87 (*c* 0.153, CHCl_3_); IR *ν*_max_ (film) 3414, 3299, 2956, 2924, 2868, 1748, 1653, 1501, 1461, 1437, 1206, 752 cm^-1^; ^1^H NMR (CDCl_3_) δ 0.65 (3H, s, H-20), 0.85 (3H, d, *J =* 5.8 Hz, H-16), 1.04 (3H, s, H-18), 1.45 (3H, s, H-17), 3.27 (4 H, m, Tript H-3), 3.62 (3H, s), 3.64 (3H, s), 4.83 (1H, m, Tript H-2), 4.94 (1H, m, Tript H-2), 6.04 (1H, d, *J =* 7.9 Hz, Tript NH), 6.10 (1H, d, *J =* 6.8 Hz, Tript NH), 6.92 (1H, d, *J =* 2Hz, Tript H-5), 6.93 (1H, d, *J =* 2 Hz, Tript H-5), 7.07 (2H, dd, *J =* 7.5, 7.5 Hz, Tript H-8), 7.15 (2H, dd, *J =* 7.5, 7.3 Hz, Tript H-9), 7.31 (2H, d, *J =* 8.0 Hz, Tript H-7), 7.50 (1H, d, *J =* 8.2 Hz, Tript H-10), 7.52 (1H, d, *J =* 8.2 Hz, Tript H-10), 8.69 (1H, s, NH); ^13^C NMR (CDCl_3_): 37.61 (t, C-1), 19.64 (t, C-2), 38.06 (t, C-3), 43.94 (s, C-4), 53.85 (d, C-5), 21.06 (t, C-6), 37.56 (t, C-7), 126.23 (s, C-8), 139.54 (s, C-9), 39.82 (s, C-10), 25.73 (t, C-11), 34.56 (t, C-12), 31.74 (d, C-13), 44.14 (t, C-14), 172.59 (s, C-15), 19.45 (q, C-16), 19.76 (q, C-17), 29.65 (q, C-18), 177.26 (s, C-19), 18.14 (q, C-20); Trypt: 172.97, 172.67 (s, C-1’); 53.17, 53.12 (d, C-2’); 27.84, 27.58 (t, C-3’); 109.90, 109.86 (s, C-4’); 123.02, 122.99 (d, C-5’); 136.39 (s, 2 C, C-6’); 111.59, 111.52 (d, C-7’); 119.54, 119.53 (d, C-8’); 122.26, 122.21 (d, C-9’); 118.45, 118.43 (d, C-10’); 127.78, 127.60 (s, C-11’); 52.40, 52.33 (q, OMe); ESI-MS *(m/z)*: 759.4151. Calcd for [C_44_H_56_N_4_O_6_Na^+^]: 759.4097.

*Labd-8(17)-en-15,19-dioic acid, 15-glycyl methyl ester amide* (**11**). The diacid (1.04 mmol) was treated with oxalyl chloride to obtain the corresponding acid chloride. Treatment of the acid chloride with the glycine methyl ester.HCl /TEA yielded the amide **11** as colourless resin (0.48 mmol, 47% yield). Treatment of the diacid (215 mg, 0.64 mmol) with DCC/DMAP afforded after treatment with the AAE.HCl and purification the compound **11** (56 mg, 0.138 mmol, 21% yield, Rf: 0.38, PE:EtOAc 1:1). [*α*]_D_^20^: +26 (*c* 1.765, CHCl_3_); IR *ν*_max_ (film) 3314, 2952, 2868, 1752, 1645, 1521, 1210, 756 cm^-1^; ^1^H NMR (CDCl_3_) δ 0.55 (3H, s, H-20), 0.85 (3H, d, *J =* 6.0 Hz, H-16), 1.17 (3H, s, H-18), 3.66 (3H, s), 3.92 (1H, dd, *J =* 18.4, 4.8 Hz, Gly H-2), 4.00 (1H, dd, *J =* 18.4, 4.8 Hz, Gly H-2), 4.40 (1H, s, H-17), 4.74 (1H, s, H-17), 5.97 (1H, t, *J =* 4.8 Hz, NH); ^13^C NMR (CDCl_3_): 38.85 (t, C-1), 19.40 (t, C-2), 38.46 (t, C-3), 45.99 (s, C-4), 56.67 (d, C-5), 21.35 (t, C-6), 39.18 (t, C-7), 147.72 (s, C-8), 56.52 (d, C-9), 40.58 (s, C-10), 26.23 (t, C-11), 36.23 (t, C-12), 31.39 (d, C-13), 43.89 (t, C-14), 173.23 (s, C-15), 20.01 (q, C-16), 107.09 (t, C-17), 28.22 (q, C-18), 170.70 (s, C-19), 13.36 (q, C-20); Gly: 172.93 (s, C’-1), 41.29 (t, C’-2); 52.44 (q, OMe); ESI-MS: 430.2655. Calcd for [C_23_H_37_NO_5_Na^+^]: 430.2570. 

*Labd-8(17)-en-15,19-dioic acid, 15-valyl methyl ester amide *(**12**). The diacid (215 mg, 0.64 mmol) was treated with DCC/DMAP and valine methyl ester.HCl to afford after the usual work-up and purification by Sephadex LH-20, the compound **12 **as a colourless resin (34 mg, 0.076 mmol, 12% yield, Rf: 0.40, PE:EtOAc 1:1). [*α*]_D_^20^: + 20 (*c* 0.74, CHCl_3_); IR *ν*_max_ (film) 3295, 2956, 2932, 2868, 1752, 1720, 1692, 1641, 1545, 1210, 1170, 756 cm^-1^; ^1^H NMR (CDCl_3_) δ 0.52 (3H, s, H-20), 0.85 (3H, d, *J =* 6.0 Hz, H-16), 0.85 (6H, d, *J =* 6.5 Hz, Val H-4, H-5), 1.16 (3H, s, H-18), 3.66 (3H, s), 4.40 (1H, s, H-17), 4.60 (1H, ddd, *J =* 13.6, 8.8, 4.8 Hz, Val H-2), 4.75 (1H, s, H-17), 5.99 (1H, d, *J = *8, NH); ^13^C NMR (CDCl_3_): 38.92 (t, C-1), 20.10 (t, C-2), 41.85 (t, C-3), 44.31 (s, C-4), 56.50 (d, C-5), 26.23 (t, C-6), 39.30 (t, C-7), 148.18 (s, C-8), 50.66 (d, C-9), 40.71 (s, C-10), 22.05 (t, C-11), 36.25 (t, C-12), 31.61 (d, C-13), 44.15 (t, C-14), 174.17 (s, C-15), 21.24 (q, C-16), 106.63 (t, C-17), 29.20 (q, C-18), 183.37 (s, C-19), 13.05 (q, C-20); Val: 172.83 (s, C’-1), 56.71 (d, C’-2), 31.61 (d, C’-3), 24.84 (q, C’-4), 23.00 (q, C’-5); 52.44 (q, OMe); ESI-MS: 472.6656. Calcd for [C_26_H_43_NO_5_Na^+^]: 472.3039. 

*Labd-8(17)-en-15,19-dioic acid, 15-leucyl methyl ester amide* (**13**). The diacid (205 mg, 0.61 mmol) was treated with DCC/DMAP and leucine methyl ester.HCl to afford after the usual work-up and purification by CC on silica gel the compound **13** as colourless resin (62 mg, 0.134 mmol, 22% yield, Rf: 0.26, PE:EtOAc 7:3). [*α*]_D_^20^: +16 (*c* 0.63, CHCl_3_); IR *ν*_max_ (film) 3303, 2952, 2928, 2864, 1748, 1716, 1645, 1541, 1437, 1202, 1162 cm^-1^; ^1^H NMR (CDCl_3_) δ 0.53 (3H, s, H-20), 0.86 (3H, d, *J = *6.0 Hz, H-16), 0.87 (6H, d, *J =* 6.0 Hz, Leu H-5 and H-6), 1.17 (3H, s, H-18), 3.67 (3H, s), 4.40 (1H, s, H-17), 4.61 (1H, ddd, *J =* 13.6, 8.8, 5.2 Hz, Leu H-2), 4.75 (1H, s, H-17), 5.93 (1H, d, *J =* 8.4 Hz, NH); ^13^C NMR (CDCl_3_): 39.34 (t, C-1), 20.13 (t, C-2), 38.22 (t, C-3), 44.33 (s, C-4), 56.74 (d, C-5), 21.28 (t, C-6), 38.94 (t, C-7), 148.23 (s, C-8), 56.53 (d, C-9), 40.74 (s, C-10), 26.26 (t, C-11), 36.23 (t, C-12), 31.65 (d, C-13), 44.22 (t, C-14), 172.73 (s, C-15), 23.02 (q, C-16), 106.66 (t, C-17), 29.22 (q, C-18), 183.05 (s, C-19), 12.99 (q, C-20); Leu: 174.10 (s, C’-1), 50.69 (d, C’-2), 41.92 (t, C’-3), 25.04 (d, C’-4), 20.05 (q, C’-5), 22.08 (q, C’-6); 52.44 (q, OMe); ESI-MS: 486.3339. Calcd for [C_27_H_45_NO_5 _Na^+^]: 486.3195. 

*Labd-8(17)-en-15,19-dioic acid, 15-prolyl methyl ester amide *(**14**). The diacid (174 mg, 0.518 mmol) was treated with oxalyl chloride and the acid chloride was reacted with proline methyl ester.HCl/TEA to afford after the usual work-up and purification by CC on RP-8 silica the compound **14** as colourless resin (22 mg, 0.049 mmol, 10% yield, Rf: 0.15, PE:EtOAc 1:1). [*α*]_D_^20^: +4 (*c* 0.022, CHCl_3_); IR *ν*_max_ (film) 3390, 2956, 2872, 1792, 1752, 1641, 1441, 1413, 1198, 1170, 995, 764 cm^-1^; ^1^H NMR (CDCl_3_) δ 0.60 (3H, s, H-20), 0.93 (3H, d, *J =* 6.4 Hz, H-16), 1.22 (3H, s, H-18), 3.46 (1H, m, Pro H-5), 3.60 (1H, m, Pro H-5), 3.69 (3H, s), 4.45 (1H, m, Pro H-2), 4.47 (1H, s, H-17), 4.80 (1H, s, H-17); ^13^C NMR (CDCl_3_): 39.21 (t, C-1), 19.99 (t, C-2), 38.51 (t, C-3), 46.03 (s, C-4), 56.72 (d, C-5), 26.27 (t, C-6), 38.91 (t, C-7), 147.83 (s, C-8), 56.55 (d, C-9), 40.62 (s, C-10), 21.44 (t, C-11), 36.52 (t, C-12), 31.11 (d, C-13), 41.59 (t, C-14), 171.83 (s, C-15), 20.26 (q, C-16), 107.19 (t, C-17), 28.27 (q, C-18), 173.29 (s, C-19), 13.55 (q, C-20); Pro: 173.15 (s, C-1’); 58.80 (d, C-2’); 29.27 (t, C-3’); 25.03 (t, C-4’); 47.42 (t, C-5’); 52.29 (q, OMe); ESI-MS: 470.1352. Calcd for [C_26_H_41_NO_5_Na^+^]: 470.2882. 

*Labd-8(17)-en-15,19-dioic acid, 15-tryptophanyl methyl ester amide* (**15**). The diacid (205 mg, 0.61 mmol) was treated with DCC/DMAP and tryptophane methyl ester.HCl to afford after the usual work-up and purification by CC on silica gel, the compound **15** as a colorless resin (58 mg, 0.108 mmol, 18% yield, Rf: 0.22, PE:EtOAc 1:1). [*α*]_D_^20^: +63 (*c* 0.032, CHCl_3_); IR *ν*_max_ (film) 3406, 3311, 2944, 2848, 1796, 1736, 1649, 1457, 1214, 999, 744 cm^-1^; ^1^H NMR (CDCl_3_) δ 0.54 (3H, s, H-20), 0.79 (3H, d, *J =* 6.2 Hz, H-16), 1.17 s (3H, s, H-18), 3.21 (2H, m, Tript H-3), 3.60 (3H, s), 4.33 (1H, s, H-17), 4.73 (1H, s, H-17), 4.91 (1H, m, Tript H-2), 5.90 (1H, d, *J =* 8.0 Hz, NH), 6.89 (1H, br s, Tript H-5), 7.00 (1H, dd, *J =* 7.4, 7.4 Hz, Tript H-8), 7.10 (1H, dd, *J =* 7.4, 7.3 Hz, Tript H-9), 7.27 (1H, d, *J =* 8.0 Hz, Tript H-7), 7.44 (1H, d, *J =* 7.8 Hz, Tript H-10), 8.28 (1H, s, NH), 10.78 (1H, s, NH); ^13^C NMR (CDCl_3_): 39.25 (t, C-1), 20.02 (t, C-2), 38.54 (t, C-3), 46.07 (s, C-4), 56.75 (d, C-5), 26.31 (t, C-6), 38.93 (t, C-7), 147.80 (s, C-8), 56.61 (d, C-9), 40.66 (s, C-10), 21.43 (t, C-11), 36.31 (t, C-12), 31.56 (d, C-13), 44.24 (t, C-14), 172.48 (s, C-15), 20.01 (q, C-16), 107.21 (t, C-17), 28.29 (q, C-18), 173.32 (s, C-19), 13.59 (q, C-20); Trypt: 172.75 (s, C-1’); 52.98 (d, C-2’); 27.95 (t, C-3’); 110.46 (s, C-4’); 122.84 (d, C-5’); 136.33 (s, C-6’); 111.47 (d, C-7’); 119.94 (d, C-8’); 122.51 (d, C-9’); 118.82 (d, C-10’); 127.96 (s, C-11’); 52.51 (q, OMe); ESI-MS: 559.3301. Calcd for [C_32_H_44_N_2_O_5_Na^+^]: 559.3250. 

*Labd-8(17)-en-15,19-dioic acid, 15,19-diglycyl methyl ester amide* (**16**). The diacid (197 mg, 0.586 mmol) was treated with oxalyl chloride and the acid chloride was reacted with glycine methyl ester.HCl/TEA to afford after the usual work-up and purification by gel permeation on Sephadex LH-20, the compound **16** as colorless resin (131 mg, 0.274 mmol, 47% yield, Rf: 0.29, PE:EtOAc 1:1). [*α*]_D_^20^: +30 (*c* 0.13, CHCl_3_); IR *ν*_max _(film) 3378, 2948, 2932, 1756, 1649, 1529, 1206, 1178, 748 cm^-1^; ^1^H NMR (CDCl_3_) δ 0.49 (3H, s, H-20), 0.86 (3H, d, *J =* 6.0 Hz, H-16), 1.13 (3H, s, H-18), 3.67 (3H, s), 3.68 (3H, s), 3.92 (2H, m, Gly H-2), 3.97 (2H, m, Gly H-2), 4.40 (1H, s, H-17), 4.75 (1H, s, H-17), 6.19 (1H, br s, NH), 6.27 (1H, br s, NH); ^13^C NMR (CDCl_3_): 39.03 (t, C-1), 20.22 (t, C-2), 38.41 (t, C-3), 44.27 (s, C-4), 56.85 (d, C-5), 21.33 (t, C-6), 39.53 (t, C-7), 147.97 (s, C-8), 56.55 (d, C-9), 40.55 (s, C-10), 26.67 (t, C-11), 36.23 (t, C-12), 31.53 (d, C-13), 43.78 (t, C-14), 173.29 (s, C-15), 19.92 (q, C-16), 106.73 (t, C-17), 30.10 (q, C-18), 177.17 (s, C-19), 12.88 (q, C-20); Gly: 170.88, 170.63 (s, C’-1), 41.43, 41.28 (t, C’-2); 52.40 (q, 2 C, OMe); ESI-MS: 501.2332. Calcd. for [C_26_H_42_N_2_O_6_Na^+^]: 501.2941. 

*Labd-8(17)-en-15,19-dioic acid, 15,19-divalyl methyl ester amide *(**17**). Some 0.392 g of the diacid (1.17 mmol) were treated with oxalyl chloride to obtain the corresponding acid chloride who was treated with valine methyl ester.HCl/TEA to afford the diamide **17** as a colorless resin (0.293 g, 0.521mmol, 44% yield, Rf: 0.52, PE:EtOAc 6:4). [*α*]_D_^20^: +34 (*c* 2.93, CHCl_3_); IR *ν*_max_ (film) 3326, 2964, 1740, 1645, 1505, 1210, 768 cm^-1^; ^1^H NMR (CDCl_3_) δ 0.59 (3H, s, H-20), 0.89 (3H, d, *J =* 6.4 Hz, H-16), 0.90 (3H, d, *J =* 6.9 Hz), 0.91 (6H, d, *J =* 6.9 Hz), 0.92 (3H, d, *J =* 6.9 Hz) (Val H-4, H-5), 1.21 (3H, s, H-18), 3.73 (3H, s), 3.74 (3H, s), 4.48 (1H, s, H-17), 4.49 (1H, dd, *J =* 8.4, 4.0 Hz, Val H-2), 4.58 (1H, dd, *J =* 8.8, 4.8 Hz, Val H-2), 4.84 (1H, s, H-17), 6.05 (1H, d, *J =* 8.4 Hz, NH), 6.13 (1H, d, *J =* 8.4 Hz, NH); ESI-MS: 585.4012. Calcd for [C_32_H_54_N_2_O_6_ Na^+^]: 585.3982. 

*Labd-8(17)-en-15,19-dioic acid, 15,19-dileucyl methyl ester amide* (**18**). Treatment of the diacid (0.378 g, 1.13 mmol) with oxalyl chloride and leucine methyl ester.HCl/TEA afforded the diamide **18** as colourless resin (229.5 mg, 0.39 mmol, 34.5 % yield, Rf 0.50, PE:EtOAc 6:4). [*α*]_D_^20^: +9.94 (*c* 0.503, CHCl_3_); IR *ν*_max_ (film) 3318, 2956, 2872, 1748, 1641, 1521, 1202, 768 cm^-1^; ^1^H NMR (CDCl_3_) δ 0.51 (3H, s, H-20), 0.86 (3H, d, *J =* 6.2 Hz, H-16), 0.88 (6H, d, *J =* 6.1 Hz, Leu H-5 and H-6), 1.12 (3H, s, H-18), 3.65 (3H, s), 3.66 (3H, s), 4.41 (1H, s, H-17), 4.51 (1H, ddd, *J =* 13.2, 8.4, 4.8 Hz, Leu H-2), 4.58 (1H, ddd, *J = *13.2, 8.8, 4.8 Hz, Leu H-2), 4.77 (1H, s, H-17), 5.87 (1H, d, *J = *8.4 Hz, NH), 5.90 (1H, d, *J =* 8.0 Hz, NH); ^13^C NMR (CDCl_3_): 39.62 (t, C-1), 20.37 (t, C-2), 38.63 (t, C-3), 44.32 (s, C-4), 56.99 (d, C-5), 21.35 (t, C-6), 39.11 (t, C-7), 147.97 (s, C-8), 56.66 (d, C-9), 40.61 (s, C-10), 26.66 (t, C-11), 36.26 (t, C-12), 31.60 (d, C-13), 44.18 (t, C-14), 172.51 (s, C-15), 20.02 (q, C-16), 106.79 (t, C-17), 30.12 (q, C-18), 176.62 (s, C-19), 13.03 (q, C-20); Leu: 173.84 (s, 2 C, C’-1); 50.72, 50.63 (d, C’-2), 41.84, 41.47 (t, C’-3), 25.16, 24.98 (d, C’-4), 22.02 (q, 2 C, C’-5), 22.97 (q, 2 C,. C’-6); 52.31, 52.23 (q, OMe); ESI-MS: 613.3647. Calcd for [C_34_H_58_N_2_O_6_Na^+^]: 613.4193.

*Labd-8(17)-en-15,19-dioic acid, 15,19-diprolyl methyl ester amide* (**19**). Some 0.400 g (1.19 mmol) of the diacid was treated with oxalyl chloride to afford the corresponding acid chloride, which was treated with proline methyl ester.HCl/TEA to obtain the diamide **19** as colourless resin (256 mg, 0.46 mmol, 38.6% yield, Rf: 0.32, PE:EtOAc 1:1). [*α*]_D_^20^: -48.4 (*c* 2.34, CHCl_3_); IR *ν*_max_ (film) 2952, 2880, 1744, 1633, 1433, 1198, 1166, 756 cm^-1^; ^1^H NMR (CDCl_3_) δ 0.53 (3H, s, H-20), 0.87 (3H, d, *J =* 6.4 Hz, H-16), 1.15 (3H, s, H-18), 3.40 (1H, m, Pro H-5), 3.52 (3H, s), 3.56 (1H, m, Pro H-5), 3.62 (3H, s), 4.39 (1H, s, H-17), 4.41 (1H, m, Pro H-2), 4.73 (1H, s, H-17); ^13^C NMR (CDCl_3_): 40.09 (t, C-1), 20.86 (t, C-2), 38.85 (t, C-3), 46.25 (s, C-4), 60.56 (d, C-5), 26.79 (t, C-6), 39.44 (t, C-7), 148.48 (s, C-8), 57.43 (d, C-9), 41.02 (s, C-10), 21.37 (t, C-11), 36.55 (t, C-12), 31.08 (d, C-13), 41.44 (t, C-14), 171.78 (s, C-15), 20.17 (q, C-16), 106.03 (t, C-17), 27.11 (q, C-18), 176.01 (s, C-19), 14.94 (q, C-20); Pro: 173.47, 172.96 (s, C-1’); 61.75, 58.66 (d, C-2’); 29.26 (t, C-3’); 24.86 (t, C-4’); 48.23, 47.27 (t, C-5’); 52.30, 51.32 (q, OMe); ESI-MS: 581.3036. Calcd for [C_32_H_50_N_2_O_6_Na^+^]: 581.3566.

*Labd-8(17)-en-15,19-dioic acid, 15,19-ditryptophanyl ethyl ester amide* (**20**). Some 0.170 g of the diacid (0.506 mmol) was treated with oxalyl chloride to afford the corresponding acid chloride, which was reacted with tryptophane ethyl ester.HCl/TEA to obtain the diamide **20** (161 mg, 0.211 mmol, 42% yield, Rf: 0.56, PE:EtOAc 1:1). [*α*]_D_^20^: +46 (*c* 0.097, CHCl_3_); IR *ν*_max_ (film) 3414, 3311, 3932, 2868, 1736, 1637, 1509, 1461, 1214, 744 cm^-1^; ^1^H NMR (CDCl_3_) δ 0.39 (3H, s, H-20), 0.83 (3H, d, *J =* 6.2 Hz, H-16), 1.02 (3H, s, H-18), 1.15 (3H, q, *J =* 6.6 Hz, Ethyl), 1.17 (3H, q, *J =* 6.6 Hz, Ethyl), 3.26 (4 H, m, Tript H-3), 4.09 (4H, m, Ethyl), 4.37 (1H, s, H-17), 4.74 (1H, s, H-17), 4.77 (1H, m, Tript H-2), 4.93 (1H, m, Tript H-2), 6.00 (1H, d, *J =* 7.9 Hz, Tript NH), 6.05 (1H, d, *J =* 6.8 Hz, Tript NH), 6.93 (1H, d, *J =* 1.9 Hz, Tript H-5), 6.95 (1H, d, *J =* 1.9 Hz, Tript H-5), 7.09 (2H, dd, *J =* 7.8, 7.1 Hz, Tript H-8), 7.16 (2H, dd, *J =* 7.5, 7.2 Hz, Tript H-9), 7.32 (2H, d, *J =* 8.1 Hz, Tript H-7), 7.51 (1H, d, *J =* 8.1 Hz, Tript H-10), 7.54 (1H, d, *J =* 8.2 Hz, Tript H-10), 8.59 (2H, brs, NH); ^13^C NMR (CDCl_3_): 39.51 (t, C-1), 20.01 (t, C-2), 38.49 (t, C-3), 44.22 (s, C-4), 56.77 (d, C-5), 26.53 (t, C-6), 39.03 (t, C-7), 148.01 (s, C-8), 56.52 (d, C-9), 40.52 (s, C-10), 21.31 (t, C-11), 36.27 (t, C-12), 31.50 (d, C-13), 44.09 (t, C-14), 172.27 (s, C-15), 20.01 (q, C-16), 106.76 (t, C-17), 30.01 (q, C-18), 176.88 (s, C-19), 12.94 (q, C-20); Trypt: 172.66, 172.47 (s, C-1’); 53.28, 53.09 (d, C-2’); 27.87, 27.51 (t, C-3’); 110.05 (s, 2 C, C-4’); 122.99, 122.91 (d, C-5’); 136.33 (s, 2 C, C-6’); 111.52, 111.41 (d, C-7’); 119.66 (d, 2 C, C-8’); 122.34, 122.24 (d, C-9’); 118.62, 118.50 (d, C-10’); 127.88, 127.63 (s, C-11’); Ethyl: 61.60, 61.48 (t; ethyl); 14.21, 14.14 (q, Ethyl); ESI-MS: 787.9840. Calc for [C_46_H_60_N_4_O_6_Na^+^]: 787.4410.

### 3.3. Ethanol/HCl-induced ulcer model in mice

The gastroprotective activity of the compounds was determined in the ethanol/HCl-induced lesion model in mice [14,15,20,21]. The purity of the tested compounds was higher than 95% by NMR analysis. Male Swiss albino mice weighing 30 ± 3 g were used. Animals were fed on certified Champion diet with free access to water under standard conditions of 12 h dark-light period, 50% relative humidity and 22 ºC room temperature. Mice were randomly distributed into groups of eight animals each and fasted for 24 h with free access to water previous to the experiment. Fasting prior to ulcerogenic assays was used because 0.2 mL of the reference antisecretory drug lansoprazole (2-[[[3-methyl-4-(2,2,2-trifluroethoxy)-2-pyridyl]methyl]sulfinyl]benzimidazole) and the amides were administered orally. To keep the animal number to a minimum, dose-response studies were performed with the leucine derivatives **13** and **18** to set the conditions for single-dose comparison of gastroprotective effect. For the selected parent compounds, three doses were used: 25, 50 and 100 mg/kg. Fifty min after oral administration of the compounds, lansoprazole (20 mg/kg) or the vehicle (12% Tween 80, 10 mL/kg), all groups were orally treated with 0.2 mL of a solution containing 60% ethanol/0.3 M HCl (ethanol/HCl) for gastric lesion induction. Animals were sacrificed by cervical dislocation 1 h after the administration of ethanol/HCl, and the stomachs excised and inflated by injection of 5% formalin (1 mL). The ulcerated stomachs were fixed in 5% formalin for 30 min and opened along the greater curvature. The length (mm) of each lesion was measured, and the lesion index expressed as the sum of the length of all lesions [14,15,20,21]. For comparison purposes, the products **1**-**5**, **7**-**12**, **14**-**16**, **19 **and **20** were assessed at a single oral dose of 25 mg/kg. The protocols were approved by the Universidad de Talca Institutional Animal Care and Use Committee, which follows the recommendations of the Canadian Council on Animal Care [23]. Tween 80 and lansoprazole (> 98% purity by HPLC) were purchased from Sigma Chemical Co. 

### 3.4. Cell culture

#### 3.4.1. MRC-5 and Hep G2 cells

Human normal lung fibroblasts MRC-5 (ATCC CCL-171) and Hep G2 cells (ATCC HB-8065) were grown as monolayers in minimum essential Eagle´s medium (MEM), with Earle's salts, 2.0 mM L-glutamine (Sigma Chemical Co.) and 2.0 g/L sodium bicarbonate (Sigma Chemical Co.), supplemented with 10% heat-inactivated fetal bovine serum (FBS), 100 IU/mL penicillin and 100 µg/mL streptomycin in a humidified incubator with 5% CO_2_ in air at 37 ºC. Cell passage was maintained between 10 and 16 for MRC-5 and between 79 and 82 for Hep G2 cells. The medium was changed every 2 days. Culture media, antibiotics and FBS were obtained from Invitrogen Corp. 

#### 3.4.2. AGS cells

Human gastric adenocarcinoma cells AGS (ATCC CRL-1739) were grown as monolayers in Ham F-12 medium containing 1.0 mM L-glutamine and 1.5 g/L sodium bicarbonate, supplemented with 10% heat-inactivated FBS, 100 IU/mL penicillin and 100 µg/mL streptomycin in a humidified incubator with 5% CO_2_ in air at 37 ºC. The cell passage was maintained between 42 and 48. The medium was changed every 2 days. 

### 3.5. Cytotoxicity assay

Confluent cultures of MRC-5, AGS and Hep G2 cells were treated with medium containing the compounds at concentrations ranging from 0 up to 1000 µM. The products were first dissolved in DMSO and then in the corresponding culture medium supplemented with 2% FBS. The final content of DMSO in the test medium and controls was 1%. Cells were exposed for 24 h to the assayed compounds. Untreated cells served as controls. Each concentration was tested in quadruplicate together with the control and repeated three times in separate experiments. At the end of the incubation, the neutral red uptake (NRU) assay was carried out to determine cell viability [20,21]. To calculate the IC_50 _values (concentration that produces a 50% inhibitory effect on the evaluated parameter) the results were transformed to percentage of controls and the IC_50 _value was graphically obtained from the dose-response curves. 

### 3.6. Statistical analysis

Results were expressed as the mean ± SD. In all experiments, statistical differences between several treatments and their respective control were determined by one-way analysis of variance (ANOVA) and when the F value was significant, post hoc differences were determined by the Dunnett's multiple comparison test. The level of significance was set at P < 0.05. All statistical analyses were performed using the software Statistica 5.1 (StatSoft, Inc.) and Statistical Package S-Plus 2000. 

## 4. Conclusions

In summary, the new compounds prepared were more active as gastroprotective agents than the C-19 monoamides prepared from imbricatolic acid, 15-acetoxyimbricatolic acid and the corresponding 8(9)-en isomers [21]. Relevant gastric lesion-preventing effects with very low cytotoxicity were found at a single oral dose of 25 mg/kg for some of the compounds. Further studies are needed to fully reveal the potential of labdane diterpenes as templates for the synthesis of new gastroprotective drugs. 
